# Successful Use of Human AB Serum to Support the Expansion of Adipose Tissue-Derived Mesenchymal Stem/Stromal Cell in a Microcarrier-Based Platform

**DOI:** 10.3389/fbioe.2020.00307

**Published:** 2020-04-15

**Authors:** Francisco Moreira, Amanda Mizukami, Lucas Eduardo Botelho de Souza, Joaquim M. S. Cabral, Cláudia L. da Silva, Dimas T. Covas, Kamilla Swiech

**Affiliations:** ^1^Department of Bioengineering, Instituto Superior Técnico, iBB-Institute for Bioengineering and Biosciences, Universidade de Lisboa, Lisbon, Portugal; ^2^Center for Cell-based Therapy, Regional Blood Center of Ribeirão Preto, University of São Paulo, São Paulo, Brazil; ^3^Department of Pharmaceutical Sciences, School of Pharmaceutical Sciences of Ribeirão Preto, University of São Paulo, São Paulo, Brazil

**Keywords:** mesenchymal stem/stromal cells, adipose tissue, xenogeneic(xeno)-free culture, AB serum, microcarriers, bioreactor

## Abstract

Mesenchymal stem/stromal cells (MSC) are promising candidates for cell-based therapies and for the promotion of tissue repair, hence the increase of clinical trials in a worldwide scale. In particular, adipose tissue-derived stem/stromal cells (AT MSC) present easy accessibility and a rather straightforward process of isolation, providing a clear advantage over other sources. The high demand of cell doses (millions of cells/kg), needed for infusion in clinical settings, requires a scalable and efficient manufacturing of AT MSC under xenogeneic(xeno)-free culture conditions. Here we describe the successful use of human AB serum [10%(v/v)] as a culture supplement, as well as coating substrate for the expansion of these cells in microcarriers using (i) a spinner flask and (ii) a 500-mL mini-bioreactor (Applikon^TM^ Biotechnology). Cells were characterized by immunophenotype and multilineage differentiation potential. Upon an initial cell adhesion in the spinner flask of 35 ± 2.5%, culture reached a maximal cell density of 2.6 ± 0.1 × 10^5^ at day 7, obtaining a 15 ± 1-fold increase. The implementation of the culture in the 500-mL mini-bioreactor presented an initial cell adhesion of 22 ± 5%, but it reached maximal cell density of 2.7 ± 0.4 × 10^5^ at day 7, obtaining a 27 ± 8-fold increase. Importantly, in both stirred systems, cells retained their immunophenotype and multilineage differentiation potential (osteo-, chondro- and adipogenic lineages). Overall, the scalability of this microcarrier-based system presented herein is of major importance for the purpose of achieving clinically meaningful cell numbers.

## Introduction

Mesenchymal stem/stromal cells (MSC), characterized as multipotent cells, have been the focus of academia, as well as cell therapy industries, due to their ability to differentiate into a variety of lineages (osteoblasts, adipocytes and chondroblasts, among others), as well as their paracrine activity which modulates inflammation and other cell processes (Caplan and Bruder, 2001; Kondo et al., 2003; Kinney et al., 2011; Mahla, 2016). Because of the low levels of expression of the major histocompatibility complex I (MHC-I), some authors consider MSC hypoimmunogenic cells more suitable for cell therapy purposes, namely in allogeneic settings (Kassem et al., 2004; Deuse et al., 2011). However, despite the controversy surrounding the definition, nomenclature and marking of “stem cell and regenerative medicine therapies,” MSC are potentially impactful in multiple diseases but there is need for further improvements (Caplan, 2017; Sipp et al., 2018).

According to International Society of Cellular Therapy (ISCT), MSC are considered a heterogeneous cell population characterized by spontaneous adherence to plastic, positivity for CD105, CD73, and CD90 and negativity for the expression of CD45, CD34, CD14 or CD11b, CD79 or CD19, and human leukocyte antigen class II, and ability to differentiate *in vitro* (osteoblasts, adipocytes and chondroblasts) (Noronha et al., 2019). MSC can be found in bone marrow (BM), adipose tissue (AT), muscle tissue and umbilical cord matrix (UCM), among others (Caplan and Bruder, 2001; Caplan, 2011). Nevertheless, cells isolated from different sources do not present exactly the same characteristics (Klingemann et al., 2008), diverging not only in cell number and proliferative capacity, but also in expression levels of different cytokines, making the choice of cell source a key feature (Musina et al., 2006).

Limitations related to the isolation procedure include low percentage of the target cells in the collected biopsy and a highly invasive collection method (BM), high number of contaminants (AT) and low yield of number of cells per unit (UCM) (Zeddou et al., 2010; Gazit et al., 2011). In particular, AT MSC can be collected in a high number (approximately 1 × 10^5^ cells per gram of tissue) compared to other sources (Ra et al., 2011) and are considered a medical waste from liposuction (i.e., a less invasive procedure compared to BM collection) which is discarded daily, therefore sidestepping any ethical problems related to the collection (Ringdén et al., 2006; Schäffler and Büchler, 2007). AT MSC presents advantages not only in the cell isolation step but also displays desirable characteristics for cellular therapy. These cells could differentiate along classical mesenchymal lineages and more recently into other cell types, including neuronal cells, cardiomyocytes, hepatocytes, pancreatic cells, suggesting multilineage plasticity across different germ layers (Mahmoudifar and Doran, 2015). Moreover, AT MSC have been demonstrated to have a superior angiogenic capacity and capable of supporting hematopoiesis (Baptista, 2020).

Although the standard process for the *ex vivo* expansion of MSC involves 2D static culture systems, typically employing fetal bovine serum (FBS) for culture medium supplementation, some efforts have been made to substitute this supplement due to the drawbacks intrinsic to the serum (Jung et al., 2012a). Besides the ethical concerns involved in blood harvesting from animals, the main limitations of FBS use refer to batch-to-batch variability, viral/prion transmission risks and potential to promote immunological reactions (Selvaggi et al., 1997; van der Valk et al., 2004; Meuleman et al., 2006). As an alternative, human serum (autologous or pooled allogeneic), platelet lysate and umbilical cord blood serum have been identified as promising FBS substitutes (Doucet et al., 2005; Schallmoser et al., 2007; Pérez-Ilzarbe et al., 2009; Stute et al., 2017). In particular, both autologous and allogeneic human serum have been successfully used for human MSC expansion, and expanded cells have maintained the expected identity, while displaying a low contamination risk since human blood components have been used in clinical practice for years (Shahdadfar et al., 2005; Bieback and Kluter, 2007; Schallmoser et al., 2007; Tan et al., 2015). In addition, human AB serum (AB HS) presents a huge advantage in terms of availability and has been proven to be an efficient FBS substitute for MSC culture, resulting in similar cumulative population doubling (dos Santos et al., 2017).

The potential use of MSC in the cellular therapy field carries a huge manufacturing challenge in order to reach a clinically relevant number of cells, estimated in 1 to 5 million cells per kilogram of patient weight (Jung et al., 2012b; dos Santos et al., 2013). None of the sources available for MSC isolation can provide this quantity of cells, turning the *ex vivo* expansion process mandatory. Establishing an automated GMP-compliant scalable bioprocess to achieve sufficient cell numbers and capable of maintaining the characteristics and function of cells in a time and cost effective manner is a major hurdle (Trainor et al., 2014). Appropriately, a bioreactor expansion process is able to reach clinical relevant cell numbers, while meeting the criteria mentioned above (dos Santos et al., 2013). This work describes the establishment of a microcarrier-based stirred-tank bioreactor, using AB HS supplemented medium, for the efficient expansion of AT MSC under xenogeneic (xeno)-free conditions, in which the cells maintained both phenotype and differentiation properties.

## Materials and Methods

### Isolation of AT MSC

Adipose tissue samples (500 mL) from liposuction (female, 30–40 years old) were provided by the Clinical Hospital (Ribeirão Preto Medical School, University of Sao Paulo), after ethical consent (Process 375415/2013). Samples were washed with Phosphate Buffered Saline Solution (PBS) and homogenized. After resting, two distinct phases were formed, an AT supernatant and an aqueous infranatant phase, the latter being discarded. After the wash, the sample was incubated with 0.1% Collagenase type II (Sigma Aldrich, St Louis, MO, United States) at 37°C for 30 min. Furthermore, the sample was incubated with Ammonium-Chloride-Potassium solution (Sigma Aldrich, St Louis, MO, United States) for 10 min to lyse red blood cells, diminishing the presence of blood contaminants. Subsequently, the highly viscous solution was subjected to a filtration step and centrifuged. The supernatant was discarded and the pellet was resuspended in alpha Minimum Essential Eagle Medium (a-MEM) (GIBCO, Grand Island, NY, United States) supplemented with 10% (v/v) Human AB serum (AB HS) [in-house produced (dos Santos et al., 2017)] and 1% (v/v) antibiotic-antimycotic (Gibco, Burlington, ON, Canada). For counting, the Trypan Blue (0.4%) (Gibco, Grand Island, NY, United States) exclusion method was used. Cells were plated in T-flasks at an initial seeding density of 5000–10000 cells/cm^2^ and expanded until passages 4–5. After culture, cells were cryopreserved in a liquid/vapor phase nitrogen tank. MSC from three independent donors were used in this work (*n* = 3).

### Cell Culture Under Static Conditions

Upon thawing, cryopreserved cells were seeded (passages 4–5) on T-flasks at a density of 3000 cells/cm^2^ in a-MEM supplemented with 10% (v/v) AB HS. After reaching 80–90% confluence, cells were detached using TrypLE^TM^ 1x (Gibco, Grand Island, NY, United States) and subcultured at the same cell density until passage 3 or 4 (P3/P4). T-flasks were maintained at 37°C under a 5% CO_2_ humidified atmosphere, with medium exchange every 3 to 4 days. The Trypan Blue (0.4%) exclusion method was used to determine cell number and viability.

### Dynamic Culture Conditions

#### AT MSC Expansion in Spinner Flasks

For the dynamic culture of AT MSC, 100 mL spinner flasks (Bellco Glass, Inc., United States) equipped with 90° paddles and a magnetic stir bar were used, with a working volume of 80 mL. Plastic microcarriers (SoloHill Engineering, Inc., United States) were coated by incubating with a-MEM + 10% (v/v) AB HS overnight. After adding 20 g/L of the pre-coated plastic microcarriers to the spinner flasks, 4 × 10^6^ cells were seeded, making a total final volume of 40 mL. For the first 24 h, a continuous stirring regimen was set at 30 rpm, and from this point onward, agitation was set at 40 rpm. At day 2 of culture, 40 mL of fresh culture medium was added to the spinner flasks, and from thereon, 25% (v/v) of culture medium was changed every day.

Cell counting was performed by removing a microcarrier-cell suspension sample of 0.5 mL from the homogeneous culture. Microcarriers were then washed with 2 mL of PBS and 1 mL of TrypLE Express 1x was added. The microcarrier suspension was then incubated at 37°C for 7 min at 650 rpm using Thermomixer (Eppendorf AG, Hamburg, Germany). Subsequently, 4 mL of a-MEM + 10% (v/v) AB HS was added to stop enzymatic activity and the cell/microcarrier suspension was filtered using a 100 mm Cell Strainer (BD Biosciences, NJ, United States). Cell number and viability were determined using the Trypan Blue exclusion method. Cell adhesion efficiency is calculated as the percentage of cells that successfully attached to the microcarrier beads after 24 h of culture (relative to day 0). MSC from three independent donors were used (*n* = 3).

#### AT MSC Expansion in the Applikon Mini-Bioreactor

A 500 mL mini-bioreactor (Applikon^TM^ Biotechnology) equipped with a three-blade pitched impeller was used. The culture parameters were set to: pH 7.3, 20% of DO by headspace aeration (N_2_, O_2_ and air) and temperature 37°C. Pre-coating of plastic microcarriers was performed as previously described (see section “AT MSC Expansion in Spinner Flasks”). Three bioreactor experiments with independent cell donors were performed (*n* = 3). For each run, 12.5 × 10^6^ cells were inoculated with 20 g/L of pre-coated plastic microcarriers in a final volume of 150 mL. At day 3 of culture, 100 mL of fresh culture medium was added to the bioreactor, and from thereon 25% (v/v) of culture medium was changed every day. Agitation was set at 85 rpm until day 2, 95 rpm until day 5 and 105 rpm from thereon after. The increase in agitation was an empirical parameterization to counterwork the increasing settling rate of the microcarriers due to a gradually higher occupancy by the cells, according to Stokes’ law (Ganzeveld et al., 1995). Two milliliters samples of culture were collected daily for cell counting and metabolite analysis by using the same protocol described for spinner flask cultures.

### Metabolite Analysis

Glucose and lactate were determined in the supernatant samples collected throughout the experiments using an automatic analyzer YSI 7100MBS (Yellow Springs Instrument, Yellow Springs, OH, United States). The analyzes are based on the specificity of immobilized enzyme electrodes for a single target analyte, allowing a rapid, accurate and largely interference free quantification. The range of measurement of glucose and lactate is 0.05–25 g/L and 0.05–2.70 g/L, respectively.

### Characterization of the Expanded Cells by Immunophenotyping

At the beginning and at the end of bioreactor culture, cells were analyzed by flow cytometry (FACSCalibur, Becton Dickinson, San Jose, CA, United States) using a panel of mouse anti-human monoclonal antibodies against CD14 (PE-conjugated), CD19 (APC-conjugated), CD31 (PE-conjugated), CD34 (PE-conjugated), CD45 [fluorescein isothiocyanate (FITC)-conjugated], CD54 (PE-conjugated), CD73 (PE-conjugated), CD80 (PE-conjugated), CD90 (PE-conjugated), CD105 (PerCP-conjugated), and HLA-DR (PerCP-conjugated) (Becton Dickinson Immunocytometry Systems, San Jose, CA, United States). A minimum of 10 000 events were collected for each sample and the data was acquired using FACSCalibur.

### Multilineage Differentiation Potential of the Expanded Cells

After expansion in the mini-bioreactor, cells were evaluated regarding their potential to differentiate into adipocytes, osteocytes and chondrocytes. For adipogenic and osteogenic differentiation, cells were plated in duplicate at a density of 3000 cells/cm^2^ on a 24-well plate (pre-coated with CELLstart, dilution 1:100) with culture medium. At confluence, cells were induced to differentiate into osteocytes and adipocytes using StemPro^TM^ Osteogenesis and Adipogenesis Differentiation Kit (Gibco, Grand Island, NY, United States), respectively. Culture medium was exchanged twice a week. Adipogenesis was observed after 14 days for lipid droplets (Sudan II-Scarlet) and osteogenesis after 21 days for mineralized bone matrix deposition (von Kossa). For chondrogenic differentiation, 1 × 10^7^ cells were plated in droplets (5 μL) on Ultra-Low attachment multi- well plates (Corning, Lowell, United States). The plate was left in the incubator for 30 min and afterward StemPro^TM^ Chondrogenesis Differentiation medium (Gibco, Grand Island, NY, United States) was added. Total medium exchange was performed two times a week for 15 days. Cells were then stained with Alcian blue (1%, Sigma- Aldrich, St Louis, MO, United States) for 2 h to assess proteoglycan synthesis (Mizukami et al., 2016).

### Statistical Analysis

Results are presented as mean ± standard deviation (SD) of the values obtained for the different cell donors. The expansion of AT MSC in spinner flasks and stirred-tank bioreactor was performed using cells from three independent donors (*n* = 3). A one-way analysis of variance (ANOVA) was used to compare data between different groups. Tukey’s *post hoc* tests were carried out to determine the differences between groups.

## Results

### Establishment of AT MSC Microcarrier-Based Culture in Spinner Flask

Previous work from our group demonstrated the low adhesion efficiency of AT MSC in microcarrier based-stirred systems when combined with commercially available serum-/xeno-free culture medium formulations (Carmelo et al., 2015; dos Santos et al., 2011). Therefore, in an attempt to overcome this limitation, different coating strategies were tested (data not shown). MSC were cultured in microcarriers coated with human fibronectin, a-MEM + 10% (v/v) AB HS or a-MEM + 20% (v/v) AB HS. Adhesion efficiency ranged from 33% for 10% AB HS (v/v), 32% for 20% AB HS (v/v) and 37% for fibronectin, resulting in a maximal cell density of 2.7 × 10^5^ (day 6), 2.3 × 10^5^ (day 6) and 2.2 × 10^5^ cells/mL (day 7), respectively. Although the culture using a fibronectin coating appears to present a slightly higher cell adhesion efficiency, it reached lower cell densities and presented a higher lag phase. Moreover, it took an extra day to reach similar maximum cell densities compared to any of the AB HS coatings. Considering these preliminary results, 10% (v/v) of AB HS was chosen for microcarrier coating for the further experiments.

**FIGURE 1:**
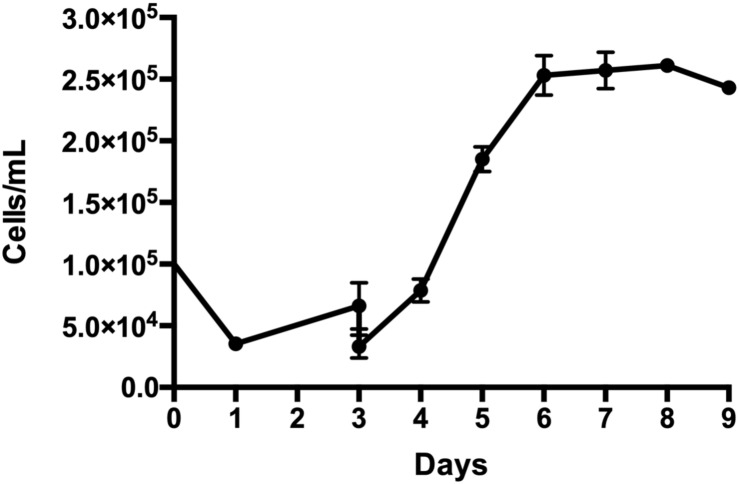
Growth profile of adipose tissue-derived stem/stromal cells (AT MSC) cultured in spinner flasks using plastic microcarrier coated with 10% (v/v) of AB HS over 9 days. Results are presented as mean ± standard error of the mean (*n* = 3, three independent donors).

Thus, AT MSC (*n* = 3, three independent donors) were cultured in spinner flasks using the microcarrier coating of 10% (v/v) of AB HS previously established. After 24 h of continuous stirring, a cell adhesion efficiency of 35 ± 2.5% was achieved, attaining a maximal cell density of 2.6 ± 0.1 × 10^5^ cells/mL at day 7, which represents a fold increase of 15 ± 1 (Figure 1). Cell viability remained above 90% during the entire cultivation.

### Scalable Stirred-Tank Bioreactor for Xeno-Free Expansion of AT MSC

**FIGURE 2:**
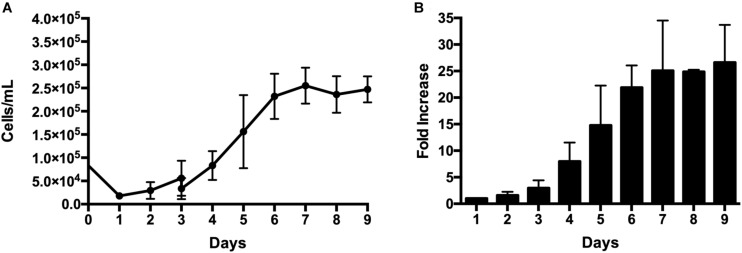
*Ex vivo* expansion of AT MSC in a 500-mL mini-bioreactor under xeno-free conditions. 12.5 × 10^6^ cells were inoculated on 20 g/L of pre-coated plastic microcarriers. Culture parameters: pH 7.3, DO = 20% and *T* = 37°C. **(A)** Cell concentration (cells/mL) over 9 days of culture. **(B)** At time points, the fold increase was calculated as the ratio between the number of cells harvested at each time-point by the number of cells harvested at day 1 (i.e., cells that successfully adhered to the microcarriers). Results are presented as mean ± standard error of the mean (*n* = 3, three independent donors).

AT MSC were then cultivated in a 500-mL stirred tank bioreactor with automated control of DO, pH, agitation and temperature. Despite using the same coating method and same initial cell density, the adhesion efficiency in the bioreactor (22 ± 5%) was lower than the one obtained for spinner flasks (Figure 2A). Although the growth profile was similar when comparing spinner flasks *versus* bioreactor, a higher cell density was achieved in the bioreactor culture at day 7 (2.7 ± 0.4 × 10^5^ cells/mL), representing a fold increase of 27 ± 8 (Figure 2B). Table 1 presents the main results obtained for the different systems and conditions tested.

**TABLE 1:** Summary of the main results obtained for the expansion of adipose tissue-derived stem/stromal cells (AT MSC) in spinner flasks and mini-bioreactor.

			**Maximal**	**Day of**	
		**Seeding**	**density cell**	**maximal**	**Fold**
**Platform**	**Coating**	**efficiency%**	**(cells/mL)**	**cell density**	**increase**
Spinner	10% AB HS	33%	2.7 × l0^5^	6	16
	20% AB HS	32%	2.3 × l0^5^	6	15
	Fibronectin	37%	2.2 × l0^5^	7	12
	10% AB HS	35 ± 2.5%	2.6 ± 0.1 × 10^5^	7	15 ± 1
Bioreactor	10% AB HS	22 ± 5%	2.7 + 0.4 × 10^5^	7	29 ± 5

**FIGURE 3:**
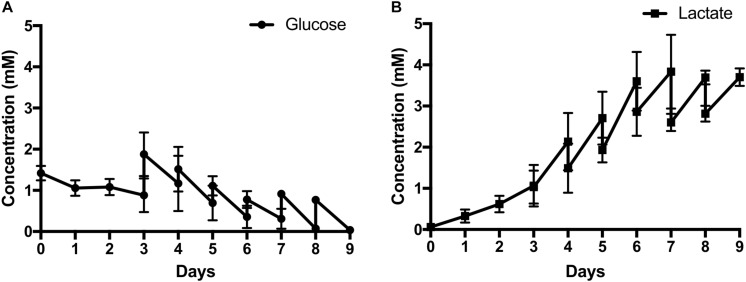
Metabolite analyses in terms of **(A)** Glucose and **(B)** Lactate concentration of AT MSC expansion on plastic microcarriers in the 500-mL mini-bioreactor using xeno-free culture medium. Results are presented as mean ± standard error of the mean (*n* = 3, three independent donors).

Glucose and lactate concentrations were measured every day throughout the culture (Figures 3A,B). As can be seen, the metabolite profile correlates with the respective growth profile with the stationary phase attained when glucose levels were nearly completely exhausted at day 8. Glucose reached 0.35 mM at day 7 and hereon after always reaching near depletion before media change. The lactate maximal level reached was 3.8 mM at day 7.

**FIGURE 4:**
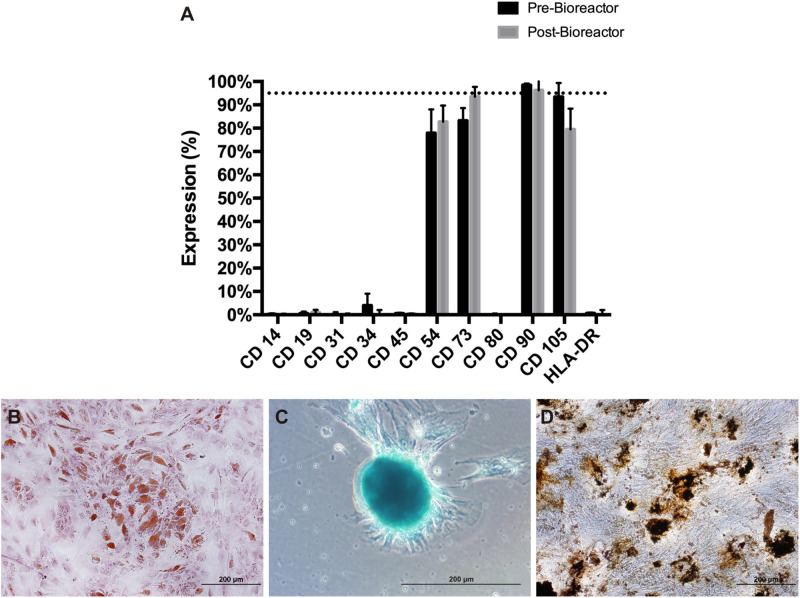
Characterization of AT MSC expanded in 500-mL mini-bioreactor. Cells harvested from plastic microcarriers were characterized through **(A)** Immunophenotyping by flow cytometry and multilineage differentiation potential for **(B)** Adipocyte progenitors/adipocytes, **(C)** Chondrocyte progenitors/chondrocytes and **(D)** Osteoblasts/osteocytes.

After cultivation, cells were enzymatically harvested and characterized regarding the immunophenotypic profile and the capacity to differentiate *in vitro* along the osteogenic, chondrogenic and adipogenic lineages. Results show that the AT MSC were positive for several markers (CD54, CD73, CD90, and CD105). Figure 4A shows that AT MSC maintained the immunophenotypic profile after expansion in the bioreactor. No significant difference was observed before and after cell expansion. Moreover, the expanded AT MSC were shown to maintain the multilineage differentiation potential (Figures 4B–D). Differentiation into adipocytes is evidenced by the formation of lipid droplets stained by Sudan II Scarlet (Figure 4B); the chondrogenic potential was attested after Alcian Blue staining, thus demonstrating the presence of proteoglycans (Figure 4C); and the osteogenic differentiation capacity by the accumulation of crystals of calcium oxalate stained with von Kossa (Figure 4D). Raw data of immunophenotype analysis by flow cytometry and metabolite results are presented in the Supplementary Material (Supplementary Table S1).

## Discussion

The most commonly used platform for human MSC expansion in the laboratory is planar culture systems, namely plastic culture flasks. While on a small scale, these systems are cost-effective and easy to handle, but in order to manufacture clinically relevant cell numbers, an unpractically large amount would be required, making it very laborious, time and cost-ineffective, as well as highly prone to contaminations (Bieback et al., 2009). In order to meet the demands of clinical settings, multi-layered cell factories have been used for MSC culture, however, despite the increase in size with a larger surface area, these retain the inherent limitations of a static planar culture (Randers-Eichhorn et al., 1996). The endeavor of establishing a GMP-compliant large-scale platform is essential to find alternatives to the standard 2D static culture systems (Sart et al., 2010). Among others, a microcarrier-based culture in a stirred bioreactor allows a better control of the various culture parameters (pH, temperature and dissolved oxygen concentration) and reducing labor. In terms of cell source, cells derived from adipose tissue are considered a valuable source for MSC isolation when compared with bone marrow (Bajek et al., 2016). Some authors, showed the ability to isolate and expand adipose-derived stromal cells in stirred bioreactor using Corning^®^ Synthemax II microcarriers (Gadelorge et al., 2017), but the majority of the studies with AT MSC are focused in tissue regeneration and its immunomodulatory properties (Zhou et al., 2011; Madonna et al., 2015). Nevertheless, reports show improvements in isolation techniques than can further optimize the process of using AT MSC in xeno-free conditions (Francis et al., 2018; Alstrup et al., 2019; Lisini et al., 2019). Although we have established a serum-free and xeno-free microcarrier-based expansion of AT MSC using a commercially available formulation (StemPro^®^ MSC SFM XenoFree) (dos Santos et al., 2011; Carmelo et al., 2015), the cell adhesion (36 ± 5.2%) and maximal cell density (1.9 × 10^5^ cells/mL) achieved were significantly inferior when compared to BM MSC expanded under the same conditions (95% and 3.6 × 10^5^ cells/mL), highlighting the need to improve the expansion process of this specific cell source under xenogeneic(xeno)-free culture conditions (Carmelo et al., 2015). Furthermore, reports show higher proliferative capacity for AT MSC than for BM MSC while retaining similar differentiation potential (Burrow et al., 2017). This prompted us to develop the present study in order to improve the culture conditions to maximize AT MSC production in this bioreactor culture system.

Based on our previous reports regarding AB HS use as culture medium supplement for MSC expansion, with cells showing similar doubling times and population doublings compared to cell cultivated with 10% (v/v) FBS supplemented culture medium (dos Santos et al., 2017; Tozetti et al., 2017), we selected this culture medium to perform this study. Thus, aiming to firstly improve the cell adhesion, three different microcarrier coatings were tested in spinner flasks: 10% (v/v) and 20% (v/v) of AB HS and fibronectin. The latter was used as a control due to its wide use as an adhesion protein for MSC cultivation (Kang et al., 2014; Bostancioglu et al., 2017).

After selecting a-MEM supplemented with 10% (v/v) AB HS as the best strategy for microcarrier coating, the adhesion efficiency calculated (35 ± 2.5%) was not higher than the one determined by Carmelo et al. (2015) (36 ± 5.2%) when using StemPro^®^ MSC SFM Xeno-Free. Despite the hindrance of not improving initial cellular adhesion, the maximal cell density achieved was significantly higher, 2.6 × 10^5^ ± 0.1 × 10^5^ when compared to 1.9 × 10^5^ cells/mL obtained in previous studies (Carmelo et al., 2015). These results indicate that human AB HS can be considered as a suitable culture medium supplement for AT MSC expansion under dynamic conditions in the spinner flask. Furthermore, using culture media to coat microcarriers instead of either commercially available coatings or higher concentrations of supplements, is more cost-effective and can reduce the handling needed for washing and removal of coating solution.

Looking for a more controlled and automated system, we implemented the culture of AT MSC in aMEM supplemented with 10% (v/v) AB HS, in the Applikon mini-bioreactor. With a tighter control over pH, dissolved oxygen, agitation and temperature, we aimed to improve even further the expansion of AT MSC under stirred conditions. Despite the observed lower adhesion efficiency (22 ± 5%) compared to the spinner flask (35 ± 2.5%), we maintained the maximal cell density attained (2.7 × 10^5^ ± 0.4 × 10^5^), resulting in a higher fold increase of 27 ± 8. We believe that improving cell adhesion would further increase maximal cell density. Not only cell-to-bead ratio is an important variable that affects cell adhesion, but also the microcarrier density plays an important role, as it promotes more cell-bead contact, hence improving adhesion. After initial microcarrier concentration, initial cell seeding density might be the next step for improving cell expansion in this platform. Moreover, one advantage of microcarrier-based culture systems is that by simply adding fresh microcarriers to the culture at a specific time point, we could overcome the surface area limitation (Panchalingam et al., 2015), without the need of sub-culturing/passaging steps.

Another key feature that must be addressed is the optimization of the feeding regimen. In the present work, 25% of medium exchange was performed at day 3 onward in order to replenish nutrients and dilute toxic metabolites (Schop et al., 2009). When adopting this feeding scheme, lactate did not reach the inhibitory levels for cell growth, recognized for human MSC culture (3.8 mM reached versus 35.4 mM reported as inhibitory) (Schop et al., 2009), but glucose reached near exhaustion levels after day 7. This nutrient limitation could be the explanation for the observed stationary phase after this period. To further maximize cell expansion in this microcarrier-based system, optimization of the culture medium feeding regimen could be implemented. An increase to 50% of culture medium exchange, increasing the change frequency to 12 h or even changing to a perfusion setting are some of the options available.

*In vitro* differentiation capacity of cells is one of the criteria proposed by ISCT to attest MSC identity after *ex vivo* culture. It is well known that MSC exhibit distinct differentiation levels according to their microenvironmental niche and culture conditions (Vishnubalaji et al., 2012). In our study, we performed this assay using a qualitative read out and it was possible to visualize the commitment of the cells with stromal lineages. However, a quantitative approach is desirable and might be a valuable tool to better characterize MSC multipotency.

## Conclusion

In conclusion, we report the establishment of a platform for AT MSC expansion under xeno-free conditions using AB HS supplement in a microcarrier-based culture system, achieving a 29-fold expansion after 7 days of culture. Although Schirmaier et al. (2014) reached 35-fold expansion at day 7 in a 2L microcarrier-based bioreactor, the media used was a serum-reduced formulation. The results presented here show the ability of AB HS formulation in expanding AT MSC under xeno-free conditions in a controlled and reproducible manner, while maintaining both phenotypic characteristics and multilineage potential.

## Data Availability Statement

The datasets generated for this study are available on request to the corresponding author.

## Author Contributions

FM carried out the experiments of AT MSC expansion and wrote the manuscript. AM helped to design, develop the experiments, and wrote the manuscript. LS and FM performed the AT MSC isolation from adipose tissue samples. JC, CS, DC, and KS supervised the project. KS helped to design the experiments. All authors discussed the results and contributed to the final manuscript.

## Conflict of Interest

The authors declare that the research was conducted in the absence of any commercial or financial relationships that could be construed as a potential conflict of interest.
