# Corrigendum: Successful Use of Human AB Serum to Support the Expansion of Adipose Tissue-Derived Mesenchymal Stem/Stromal Cell in a Microcarrier-Based Platform

**DOI:** 10.3389/fbioe.2020.594582

**Published:** 2020-10-07

**Authors:** Francisco Moreira, Amanda Mizukami, Lucas Eduardo Botelho de Souza, Joaquim M. S. Cabral, Cláudia L. da Silva, Dimas T. Covas, Kamilla Swiech

**Affiliations:** ^1^Department of Bioengineering, Instituto Superior Técnico, iBB-Institute for Bioengineering and Biosciences, Universidade de Lisboa, Lisbon, Portugal; ^2^Center for Cell-based Therapy, Regional Blood Center of Ribeirão Preto, University of São Paulo, São Paulo, Brazil; ^3^Department of Pharmaceutical Sciences, School of Pharmaceutical Sciences of Ribeirão Preto, University of São Paulo, São Paulo, Brazil

**Keywords:** mesenchymal stem/stromal cells, adipose tissue, xenogeneic(xeno)-free culture, AB serum, microcarriers, bioreactor

In the published article, the affiliation of Joaquim M.S. Cabral and Cláudia L. da Silva is incorrect. The correct affiliation for both authors is:

^1^Department of Bioengineering, Instituto Superior Técnico, iBB-Institute for Bioengineering and Biosciences, Universidade de Lisboa, Lisbon, Portugal

The authors apologize for this error and state that this does not change the scientific conclusions of the article in any way. The original article has been updated.

