# Supplementary figures and images for: Successful Use of Human AB Serum to Support the Expansion of Adipose Tissue-Derived Mesenchymal Stem/Stromal Cell in a Microcarrier-Based Platform
Source: Front Bioeng Biotechnol. 2020 Apr 15;8:307. doi: 10.3389/fbioe.2020.00307 (PMC7184110; doi:10.3389/fbioe.2020.00307)

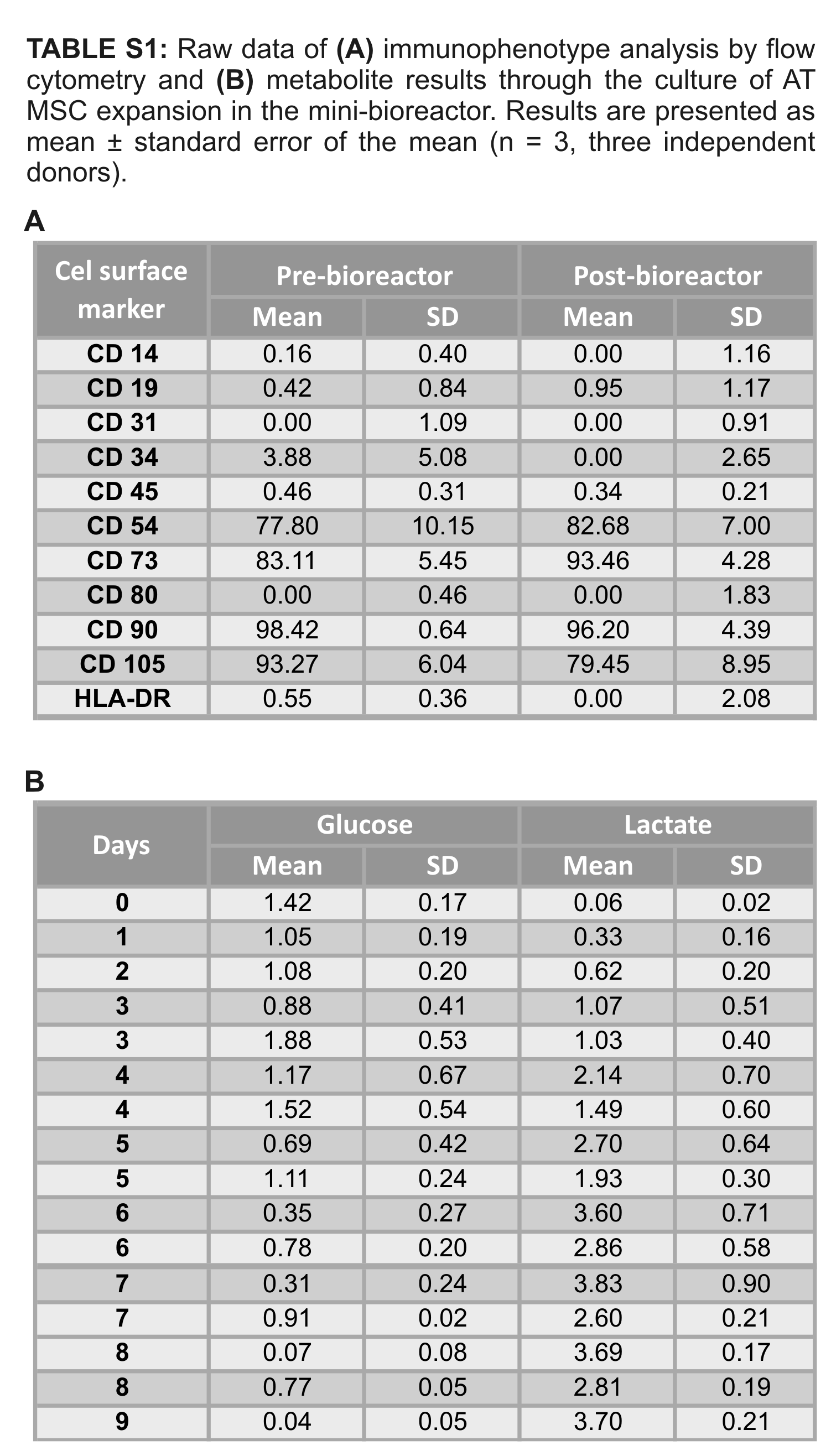

Supplement: Supplementary file 1 [file Image_1.tiff]
